# Stepwise Assembly of Fibrinogen Is Assisted by the Endoplasmic Reticulum Lectin-Chaperone System in HepG2 Cells

**DOI:** 10.1371/journal.pone.0074580

**Published:** 2013-09-10

**Authors:** Taku Tamura, Seisuke Arai, Hisao Nagaya, Jun Mizuguchi, Ikuo Wada

**Affiliations:** 1 Department of Cell Science, Institute of Biomedical Sciences, Fukushima Medical University, Fukushima, Japan; 2 Core Research for Evolutional Science and Technology, Japan Science and Technology Agency (JST), Tokyo, Japan; 3 The Chemo-Sero-Therapeutic Research Institute (Kaketsuken), Kumamoto, Japan; International Centre for Genetic Engineering and Biotechnology, Italy

## Abstract

The endoplasmic reticulum (ER) plays essential roles in protein folding and assembly of secretory proteins. ER-resident molecular chaperones and related enzymes assist in protein maturation by co-operated interactions and modifications. However, the folding/assembly of multimeric proteins is not well understood. Here, we show that the maturation of fibrinogen, a hexameric secretory protein (two trimers from α, β and γ subunits), occurs in a stepwise manner. The αγ complex, a precursor for the trimer, is retained in the ER by lectin-like chaperones, and the β subunit is incorporated into the αγ complex immediately after translation. ERp57, a protein disulfide isomerase homologue, is involved in the hexamer formation from two trimers. Our results indicate that the fibrinogen hexamer is formed sequentially, rather than simultaneously, using kinetic pause by lectin chaperones. This study provides a novel insight into the assembly of most abundant multi-subunit secretory proteins.

## Introduction

Proper folding of secretory proteins in the endoplasmic reticulum (ER) is an essential process for cellular homeostasis. Proteins in the secretory pathway are co-translationally inserted into the ER, where various reactions including disulfide bond formation, subunit assembly and N-linked glycan dependent interaction are initiated. Proteins in the secretory pathway are co-translationally inserted into the ER. The stabilization processes of immature polypeptides, including disulfide bond formation, subunit assembly and N-linked glycan dependent interaction, are largely assisted by the transient association of ER-resident chaperones and folding enzymes. They are allowed to exit the ER only after acquisition of proper conformation. The accumulation of cargo proteins that fail to exit the ER triggers sets of gene expression to adapt cells to the stress by augmentation of a set of proteins involving structural editing or enhancement of processes leading to the proteasomal degradation. These series of cellular responses to relieve ER stress are collectively called the “unfolded protein response” (UPR) [1]. These mechanisms have been designated as the ER quality control systems [2].

Calnexin (CNX), an ER-resident type I membrane protein, and its soluble homologue calreticulin (CRT), are lectin-type chaperones in that they recognize only the monoglucosylated N-linked glycans [3]. CNX and CRT assist immature protein folding by recruiting ERp57, a member of the protein disulfide isomerase (PDI) family, which facilitates the disulfide bond formation and exchange of substrate proteins [3], [4], [5]. Association-dissociation cycle of substrates with CNX/CRT is strictly regulated by the terminal glucose trimming by glucosidase II, and reglucosylation by UDP-glucose: glycoprotein glucosyltransferase 1 (UGGT1). The contribution of this cycle to the oxidative folding in the ER has been extensively studied with rather simple model proteins such as influenza virus envelopes and transferrin [6], [7]. Studies have been extended to oligomeric proteins including MHC class I and insulin receptor [8]–[10], thereby revealing these complicated processes.

Fibrinogen is a central factor in the coagulation pathway in which secreted fibrinogen is processed by thrombin and formed fibrin polymer to build fibrin clots in blood vessels [11], [12]. The mature form of fibrinogen is produced by a covalently linked hexamer composed of two sets of symmetrical trimers (α, β and γ subunits) and contains 29 intramolecular disulfide bonds but not the free thiol group [13]. Both β and γ subunits are singularly N-glycosylated whereas the α subunit is non-glycosylated. The assembly process of the hexamer has been postulated as follows. Firstly, the trimer is generated by integrating a β chain into the αγ complex, or an α chain into the βγ complex. Next, the two trimers are joined at their N-termini and form the hexamer. Participation of the ER resident chaperones in the fibrinogen folding pathway has been suggested from the results of transcription-translation system [14] and the stable expression of fibrinogen subunits by the baby hamster kidney cell lines [15]. However, the details of this chaperone-assisted mechanism in the ER remain unclear.

To explore the functional relationship of the ER lectin chaperone system and fibrinogen assembly, we examined the fibrinogen maturation process using a HepG2 cell line. Our findings indicate that the fibrinogen hexamer assembly is processed in a step-by-step mode, rather than simultaneous assembly, and the lectin chaperones function to retain the preceding complex in the ER to prevent them from secretion. The analysis revealed that dimerization of the two trimers seems to be the rate-limiting step of the hexamer formation where ERp57 assists this assembly. Our results suggest that the ER lectin chaperone system coordinates the stepwise assembly of a fibrinogen oligomer by generating a kinetic pause.

## Results

### Assembly of Fibrinogen Trimer and Hexamer Through the αγ Precursor

**Figure 1:**
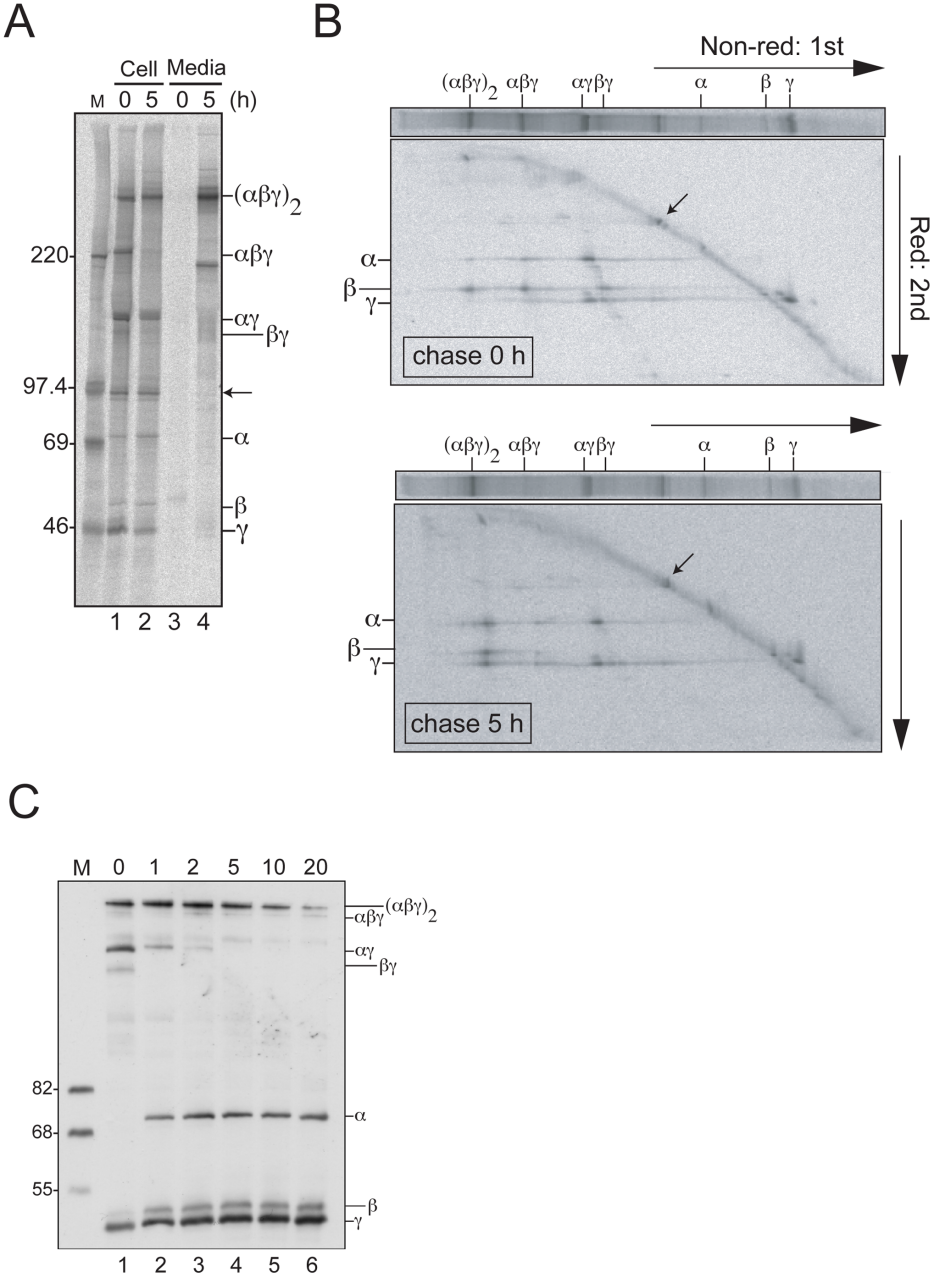
Stepwise assembly of fibrinogen in HepG2 cells. (A) Cys and Met-starved HepG2 cells were pulse-labeled for 20 min and chased for 0 or 5 hr. Cells were lysed and media were supplemented with TX-100 (final 0.5%) then immunoprecipitated using anti-fibrinogen antibody after prewash with Pansorbin cells. Immunoprecipitated samples were resolved with 4–15% SDS-PAGE in non-reducing conditions. The molecular mass of marker proteins (lane M) is shown on the left in kDa. Band positions of fibrinogen molecules are indicated on the right: (αβγ)_2_ and αβγ represent fibrinogen hexamer and trimer, respectively. Lane numbers are shown at the bottom. (B) Separated fibrinogen molecules of 0 or 5 hr chase with non-reduced SDS-PAGE in 4–15% gradient gel (horizontal axis), as described in A, were resolved by 9% SDS-PAGE after denaturing with β-mercaptoethanol (vertical axis). Of note, a 97 kDa endogenous protein, which associates with fibrinogen, is represented by an arrow in A and B. (C) HepG2 cells were exposed to the indicated concentration of DTT (mM, at the top) in culture medium for 10 min at 37°C. After alkylation of the free thiol group with IAA, the fibrinogen proteins were separated by non-reducing 9% SDS-PAGE and detected by immunoblotting using anti-fibrinogen antibody.

To understand how the mature fibrinogen heterooligomeric hexamer (αβγ)_2_ is generated in cells, we scrutinized the biogenesis in HepG2 cells. This human hepatoma cell line highly expresses all fibrinogen subunits and secretes the mature molecule into the culture medium [16], [17]. To analyze the assembly pathway of the fibrinogen hexamer with newly synthesized subunits, pulse-labeled and chased proteins were recovered by immunoprecipitation with anti-fibrinogen antibody from cell lysates and then visualized by 4–15% gradient SDS-PAGE under non-reducing conditions (Fig. 1A). Several high molecular weight bands in addition to the bands corresponding to the monomeric subunits (α, β and γ) were detected when cells were pulse-labeled for 20 min (Fig. 1A, lane 1). Essentially, after the 5 hr chase the same set of bands was observed, except for the disappearance of a band slightly larger than 220 kDa (Fig. 1A, lane 2). From the apparent molecular weight, this band was suggested to be αβγ, a trimeric complex of the α, β and γ chains. Similarly, the largest band was considered to be the mature form, hetero-oligomeric hexamer, (αβγ)_2_, and the major band between the 220 kDa and 97.4 kDa bands was to be the αγ complex. We also assumed that the faint band that migrated slightly faster than the αγ band, which disappeared after the 5 h chase, was the βγ complex.

To study whether oligomers are assembled from the precursor or gathered in only newly synthesized proteins, we conducted a two-dimensional gel electrophoresis: non-reducing SDS-PAGE for the first dimension and reducing condition for the second run. The results shown in Fig. 1B confirmed the subunit composition of the oligomers, i.e., the band assigned as (αβγ)_2_ or αβγ contained all three subunits, and the αγ band was indeed composed of α and γ subunits. The band migrating slightly faster than the αγ complex appeared to contain β and γ as assumed (chase 0 h). At the 5 h chase, the hexamer was composed of the three individual pulse-labeled subunits while the hexamer band at the pulse period (chase 0 h) contained only trace amounts of the α and γ chains (Fig. 1B). This is consistent with the previous observation where the hexamer is formed by incorporating a newly synthesized β subunit into the pre-existed αγ complex [18]. The intensity and composition of the αγ complex remained almost constant, irrespective of the 5 h chase. This observation suggests that the αγ is a stable complex and the turnover is slow. In this experimental condition, the majority of the secreted form of fibrinogen is the hexamer, while monomeric subunits were only faintly detected (Fig. 1A, lane 4). In contrast, examination of the steady-state expression indicated that monomeric forms are also stable and abundantly exist in cells (Fig. 1C and Fig. S1). While it is possible that the latter is the result of N-glycan mediated quality control as suggested by Roy S. et al [14], we found that the 97 kDa protein was consistently revealed by the immunoprecipitation experiments (Fig. 1A, lanes 1 and 2 and Fig. 1B). We speculated that this protein may participate in fibrinogen maturation since the band was only found in the intracellular fibrinogen but not in the secreted form (compare Fig. 1A lane 2 and 4). We identified this protein as Triadin, an N-glycosylated and ER-resident protein, by mass-spectrum analysis. However, we obtained no positive evidence proving its involvement (data not shown).

### Structural Stability of Fibrinogen Complexes

To understand the folding stability of the αγ complex, resistance to DTT of the disulfide bonds was assessed. We first confirmed that the anti-fibrinogen antibody used in this study actually recognized all fibrinogen subunits by immunoblotting experiments (Fig. S1A and S1B). We then exposed the cells to various concentrations of DTT for 10 min at 37°C and the thiol groups were trapped by treatment with iodoacetamide. The polypeptides were resolved by non-reducing SDS-PAGE. The immunoblot showed that the αγ complex was most sensitive to DTT, while the covalent hexamer showed marked resistance to DTT (Fig. 1C). These results suggest that the αγ complex is susceptible to disulfide exchange, compared to the fully folded hexamer.

### Intracellular Fibrinogen Molecules are Mostly Retained in the ER

**Figure 2:**
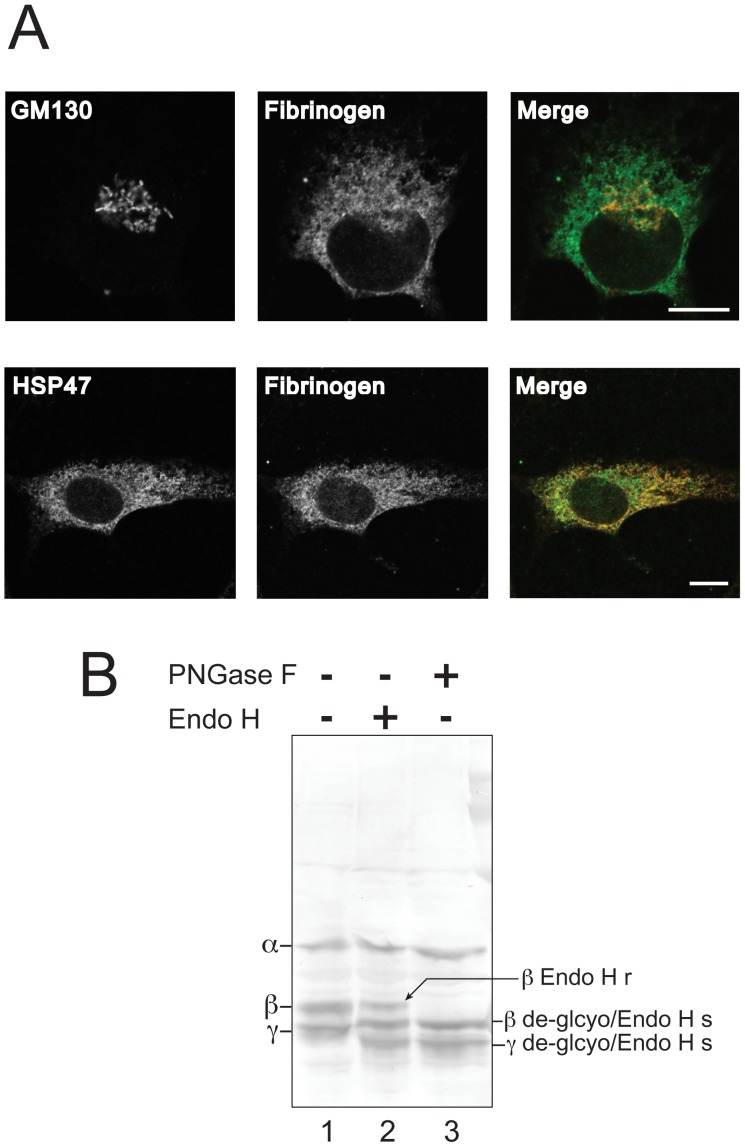
Fibrinogen molecules mainly localize at the early secretory pathway in HepG2 cells. (A) HepG2 cells split onto coverslips in 35 mm dishes were immunostained with anti-fibrinogen antibody (green) and then with anti-GM130 or HSP47 antibody (red), as described in the Materials and Methods. Scale bars represent 10 µm. (B) HepG2 cells were lysed in the lysis buffer (50 mM Hepes pH 7.4 containing 100 mM NaCl, 1 mM EDTA, 5% glycerol and 1% TX-100) and protein concentration was measured by standard Bradford assay. After denaturing with 0.5% SDS, samples were treated with PNGase F (lane 3) or Endoglycosidase H (Endo H, lane 2) according to the manufacturer’s protocol. Samples were separated with 9% SDS-PAGE (loaded 10 µg in each lane) under reducing conditions, and fibrinogen subunits were visualized by immunoblotting using anti-fibrinogen antibody. Migration of the undigested fibrinogen subunits is marked on the left. Positions of deglycosylated or Endo H sensitive (Endo Hs) or resistant (Endo Hr) forms of β or γ subunits are indicated on the right.

To validate whether the αγ complex is actually retained in the ER to serve as the reserve, we analyzed the intracellular localization of the fibrinogen molecules. Indirect immunostaining of fibrinogen, using the polyclonal antibody that recognizes all fibrinogen molecules (Fig. S1), exhibited a typical reticular pattern in the cytoplasm (Fig. 2A). Poor co-localization with Golgi marker protein GM130 was observed (Fig. 2A, top panels) whereas a soluble ER-resident protein, Heat shock protein 47 (HSP47), exhibited a similar pattern to the fibrinogen staining (Fig. 2A, bottom panels). This suggests that the fibrinogen molecules are almost all retained in the ER of HepG2 cells at steady state condition. Furthermore, N-glycans of fibrinogen β and γ subunits showed only partial resistance to Endoglycosidase H, which digests only ER form N-glycan (Fig. 2B, lane 2). Together, these results indicate that the majority of fibrinogen molecules are confined to the early secretory pathway of HepG2 cells.

### The Lectin Chaperone System Supports Retention of the αγ Complex in the ER Through Mono-glucose N-glycans

**Figure 3:**
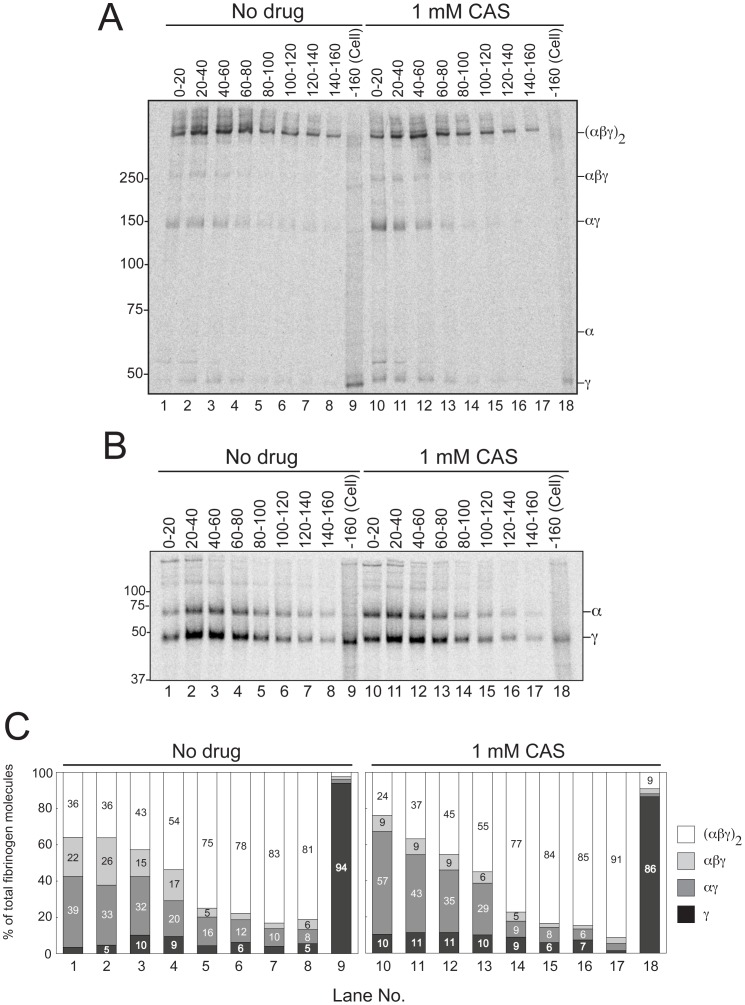
Cellular retention of fibrinogen αγ complex by ER lectin chaperone through monoglucosylated N-glycans. HepG2 cells were starved for Met and Cys and then pulse-labeled for 20 min. In the chase experiment, the medium was replaced with non-radiolabeled media every 20 min. These experiments were conducted in the presence or absence of 1 mM CAS. Samples were immunoprecipitated using anti-fibrinogen antibody and then resolved by a 4–15% gradient gel under non-reducing conditions (A), or by a 9% gel under reducing conditions (B). The lane “−160 (Cell)” denotes the immunoprecipitation samples of cell lysate after chase for 160 min. (C) Quantification of each fibrinogen molecule in A. The percentage of each fibrinogen band relative to the sum of all species (100%) is noted inside the bars. Lane numbers are shown at the bottom. White, light gray, dark gray and black bars from the top to the bottom are represented as (αβγ)_2_, αβγ, αγ and γ chains, respectively. Of note, the bands not reaching 5% intensity are not stated in the bars.

We next addressed whether the αγ complex was selectively retained in the ER. CNX preferentially binds to the monoglucosylated proteins that are regenerated through the CNX cycle until UGGT loses its ability to bind [19]. It has been reported that CNX interacts with fibrinogen [14], although the details of the maturation remain unresolved. Since the γ subunit is singly N-glycosylated, we examined the effects of castanospermine (CAS), which inhibits ER glucosidase I and II, to impede the interaction of CNX and CRT with the αγ intermediate. When examined by pulse-chase experiments, we found that secretion of the αγ complex was markedly enhanced by the CAS treatment (Fig. 3A and 3B, compare lanes 1–3 with 10–12). Quantification of each band indicated that, especially in the early time points, CAS enhances the secretion of the αγ complex and γ subunit; at the 0–20 min chase, secretion of the αγ complex was raised from 39 to 57% of the total secreted fibrinogen by 1 mM CAS treatment (Fig 3C, compare lane 1 with 10). Though we could not reveal the direct interaction of CNX/CRT and fibrinogen by sequential immunoprecipitation (data not shown), which we think was due to the short and transient interactions, the pulse-chase experiments clearly indicate the participation of the lectin chaperones in fibrinogen maturation through the association with the αγ complex. Considering that the αγ complex is relatively unstable compared with the trimer or the hexamer (Fig. 1C), CNX/CRT functions to retain the αγ complex as they expose motif(s) that UGGT recognizes.

### ERp57 Transiently Associates with the Fibrinogen Trimer to Assemble the Hexamer

**Figure 4:**
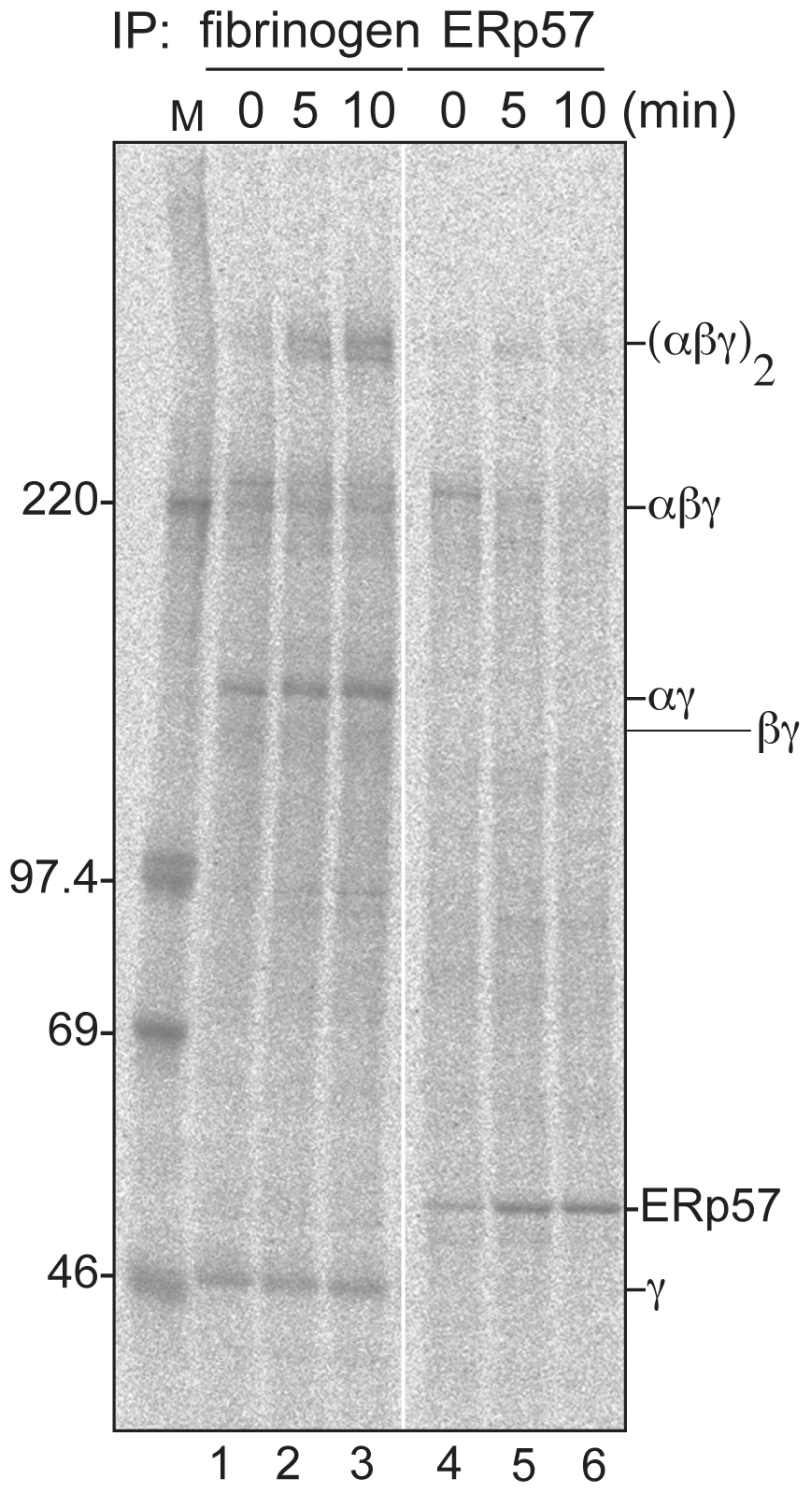
Transient binding of ERp57 to fibrinogen trimer and hexamer during the assembly. Cys and Met starved HepG2 cells were pulse-labeled for 3 min and then chased for 0, 5 and 10 min. The cell lysate was divided into two aliquots and immunoprecipitated using anti-fibrinogen (lanes 1–3) or anti-ERp57 (lanes 4–6) antibodies. Samples were separated with 4–15% gradient SDS-PAGE under non-reducing conditions and visualized with BAS-2000.

When the β chain was inserted into the preformed αγ complex, two types of hetero-oligomers, αβγ and (αβγ)_2_, were immediately formed within 20 min (Fig. 1A and 1B). To examine the details of the hexamer formation, we shortened the pulse period to 3 min so that only one round of translation was completed. After 3 min synthesis, the αβγ complex was formed whereas the signal of the (αβγ)_2_ complex hardly appeared (Fig. 4, lane 1). The hexamer was detected at the 5 min chase (Fig. 4, lane 2) and increased in the next chase period of 5 min while the trimer was decreased upon chase (Fig. 4, lane 2 and 3), indicating that the trimer was formed nearly co-translationally before the two trimers were integrated into the hexamer.

Since CNX is known to function as a scaffold for ERp57 [20]–[23] and the αγ complex appeared to be retained in the ER by CNX (Fig. 3), we examined whether ERp57 was associated with fibrinogen intermediates. We found that, at pulse-label 3 min and 0 min chase, ERp57 binds to the trimer, but not to the αγ complex or the hexamer (Fig. 4, lane 4). Upon further chase, the trimer was dissociated from ERp57, whereas the hexamer was recovered in the ERp57 co-immunoprecipitates (Fig. 4, lane 4–6). Sequential immunoprecipitation revealed that the target of ERp57 in the biogenesis of fibrinogen was almost exclusively the trimer (Fig. S2). These results suggest that the role of ERp57 in fibrinogen hexamer assembly is to integrate two trimers into the hexamer.

### ERp57 Accelerates Fibrinogen Oligomer Formation in a Reglucosylation-independent Manner

**Figure 5:**
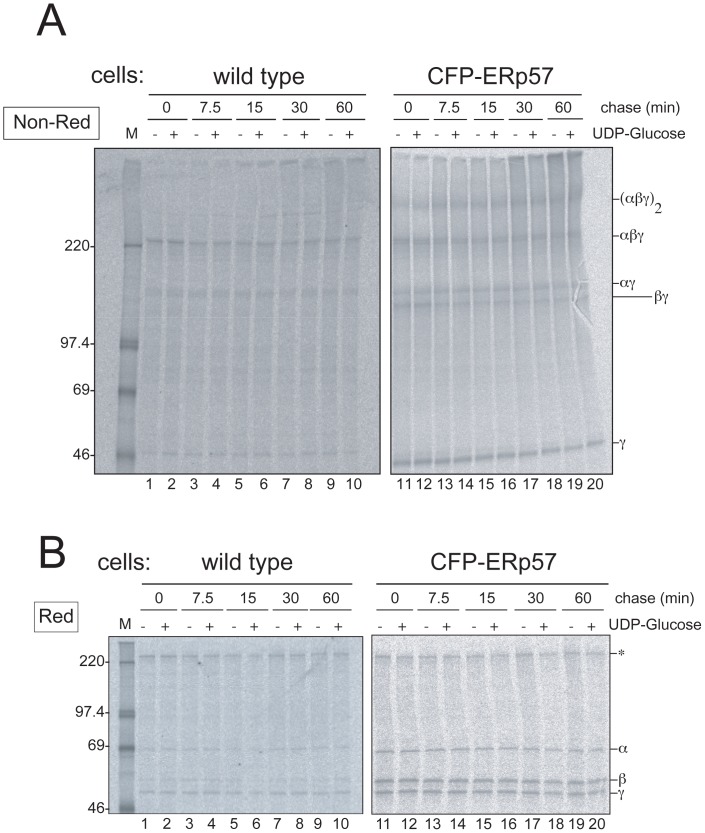
Acceleration of fibrinogen assembly by ERp57 in a reglucosylation-independent manner. Wild type or CFP-ERp57 stably overexpressing HepG2 cells were starved for Met and Cys for 3 hr. After pulse labeling for 5 min, the microsomes were prepared at 4°C and then suspended in the cytosolic condition buffer [8]. Refolding reaction was initiated by incubation at 37°C for the indicated times (min) in the presence (+) or absence (-) of 1 mM UDP-glucose. After immunoprecipitation using anti-fibrinogen antibody, the samples were separated by a non-reducing SDS-PAGE in a 4–15% gel (A) or reducing SDS-PAGE in a 9% gel (B). The molecular mass of marker proteins is shown on the left and each fibrinogen subunit is indicated on the right. The asterisk represents a non-specific band derived from Pansorbin cells.

To further ascertain the contribution of ERp57 to fibrinogen maturation, we generated HepG2 cells stably expressing CFP fused ERp57 and examined the fibrinogen maturation rate by using “pulse-labeled microsomes”, which monitors protein folding/assembly in microsomes supplemented with UDP-glucose to facilitate CNX/CRT association with N-glycans [7]. After 5 min pulse-labeling, the cells were cooled on ice and homogenized to prepare the microsomes. Folding was restarted at 37°C with or without UDP-glucose, which is transferred to deglucosylated N-linked glycan as glucose by UGGT1 [24]. Conversion of the fibrinogen trimers to the hexamer was observed in the microsomes from wild type cells at 7.5 min and increased (Fig. 5A, lanes 3 and 4). These kinetics resemble transferrin folding [7]. In contrast, CFP-ERp57 overexpressing cells produced the hexamer immediately after the chase (Fig. 5A, lanes 11–12) and gradually increased during the chase, consistent with Fig. 4, indicating ERp57 involvement early on. Interestingly, CFP-ERp57 overexpression caused βγ complex formation, which was rarely observed in wild type cells. The βγ complex was decreased during incubation, suggesting that a higher oligomer was formed from the complex or dissociated to the monomeric subunit. Unlike transferrin [7], the addition of UDP-glucose to the incubation mixture had no effects in the oligomer formation (compare UDP-glucose - and+lanes) even in the CFP-ERp57 overexpressing condition. These results suggest that CNX/CRT binding does not play a major role in fibrinogen oligomer assembly and that ERp57 works independently from CNX/CRT scaffolding, alike the case of major histocompatibility class I chain assembly [25].

### Knockdown of ERp57 Suppresses the Final Step of Fibrinogen Assembly

**Figure 6:**
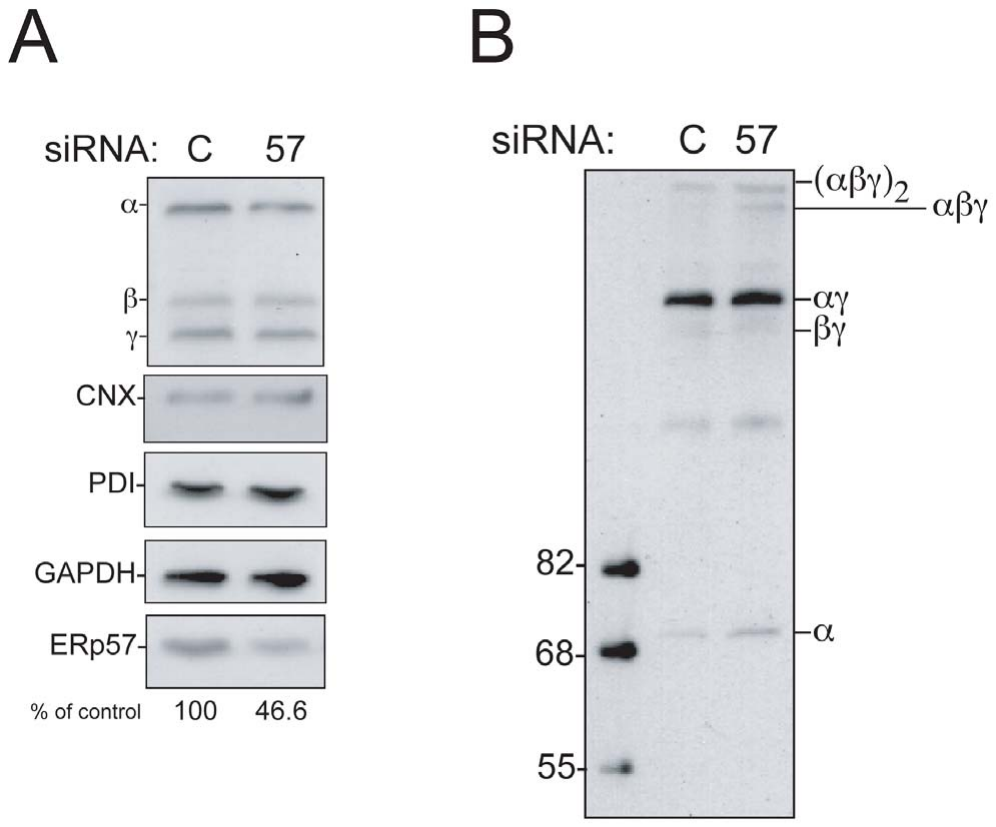
Down regulation of ERp57 retards fibrinogen hexamer formation. (A) HepG2 cells were transfected with indicated double strand RNA (C, control: 57, ERp57) for 96 hr. Cells were lysed in SDS-PAGE sample buffer and subjected to a reducing 9% SDS-PAGE. Proteins were visualized using indicated antibodies. Knock down effects of ERp57 are quantitated using ImageJ and shown at the bottom of panel A versus GAPDH. (B) HepG2 cells were transfected as described in A, and fibrinogen molecules were detected by western blotting using anti-fibrinogen antibody in non-reducing conditions.

To further prove the responsibility of ERp57 in hexamer formation, we reduced the expression level of ERp57 by RNAi. As shown in Fig. 6A, treatment of cells with ERp57 siRNA specifically decreased the amount of ERp57 to 46.6% while CNX, PDI and GAPDH were not affected. Immunoblotting of the fibrinogen chains resolved by non-reducing SDS-PAGE revealed that the amount of trimer was clearly increased by the ERp57 siRNA treatment compared to the control (Fig. 6B). This result suggests that ERp57 down-regulation delays the conversion of the trimer to the hexamer and supports the notion of the positive role of ERp57 in the fibrinogen maturation.

## Discussion

**Figure 7:**
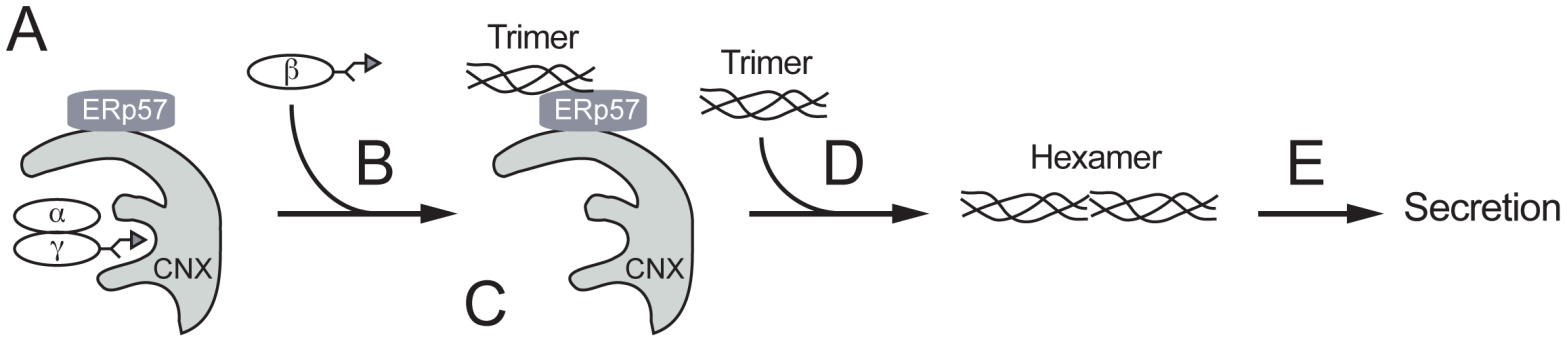
A working model of ER chaperone assistance in fibrinogen assembly. (A) Calnexin (CNX) holds the fibrinogen αγ complex through monoglucosylated N-linked glycans. (B) The newly synthesized fibrinogen β chain is integrated into the αγ complex. (C) A fibrinogen trimer is formed and handed off to ERp57 from CNX. (D) ERp57 facilitates the integration of the two trimers into the hexamer. (E) The properly assembled fibrinogen hexamer is moved forward to the later secretory pathway. Noteworthy, N-glycosylated β and γ chains are illustrated by the Y motif.

The assembly of multisubunit proteins requires complicated steps because of the generation of various folding intermediates [26], [27]. A folding intermediate needs to be kept unassembled, however it should not be recognized as terminally misfolded by the disposal machinery. Molecular chaperones should prevent immature proteins from undesired interactions. To understand how the ER chaperone system assists the assembly of large oligomeric proteins, we focused on fibrinogen, one of the most abundant secretory proteins in human blood. Our analyses showed that fibrinogen maturation involves two steps; integration of β chain into the pre-formed αγ complex to form the trimer, and secondly, integration of the two trimers to assemble the hexamer (see our working model in Fig. 7). The ER-resident lectin chaperones, CNX and CRT, retain the αγ complex, through monoglucosylated N-linked glycans (Fig. 7A based on Fig. 1 and 4). Newly synthesized β chains appeared to be incorporated into the αγ complex immediately (Fig. 7B from Fig 1B). Once the trimer is formed, the conversion to the hexamer is completed within 10 min (Fig. 7C and D). Our results also suggest that ERp57 is responsible for the final assembly process of the two fibrinogen trimers to the hexamer (Fig. 4, lanes 4–6, Fig. 6B).

The detailed analysis suggests that the trimer/hexamer is not generated directly by assembly of individual subunits (Figs 1 and 3). Since disruption of the αγ complex by DTT treatment during pulse-labeling generates high molecular weight aggregates (our unpublished results), precursor-mediated assembly is a prerequisite for proper fibrinogen maturation. As shown in Figs. 1C and 3, the αγ complex is retained in the ER. Since both the α and γ subunits do not contain an ER retention motif such as KDEL, and the prevention of glucose trimming enhanced secretion of the hetero-dimer (Fig. 2A), continuous interaction with CNX/CRT should be important for retention to prevent premature secretion of the intermediates. It is likely that the dimer is the substrate of UGGT1 to maintain CNX/CRT interaction but not by the disposal machinery. Since CAS treatment did not inhibit the assembly of the fibrinogen hexamer (Fig. 3A and Fig. 5A), the integration of the β chain into the αγ complex is independent of the lectin activity of CNX and CRT.

In many reports on fibrinogen assembly, the αγ complexes are formed in most assay systems such as HepG2 cells, stably expressing COS7 or baby hamster kidney cell lines in combinations of the fibrinogen three subunits [14], [16], [17], [28], [29]. Although the αγ complex was suggested to be insignificant and generated from a pool of residual chains [17], our present data indicate that the αγ complex is an actual precursor of the fibrinogen trimer that deploys in the ER beforehand. Since the binding of the newly translated α chain into the preformed βγ was detected very slightly (Fig. 1B), we suppose that the βγ complex is a bi-product of assembly that is only produced when a balance of chaperone constituents is disturbed. The overproduction of the βγ subunit was observed in pulse-labeled microsomes of CFP-ERp57 overexpressing cells (Fig. 5) or in stable overexpression of Ero1α or β, both of which oxidize PDI by electron transfer and contribute to maintain the ER oxidative condition [30], in HepG2 cell lines (our unpublished results). Overexpression of Ero1α or β oxidizes PDI [31]–[33] and ERp57 [34] at which disulfide bond formation of substrate proteins is enhanced. In the least, the current observation indicates that the modes of assembly are largely affected by the oxidation/thiol-exchanging activity of the ER.

The final step of fibrinogen hexamer assembly that followed immediately after the formation of the trimer could be regarded as the rate-limiting step of fibrinogen maturation (Fig. 5). The pull-down assay using anti-ERp57 antibody clearly indicated that ERp57 is involved in hexamer assembly through the transient association and dissociation with the trimer. Herein, we present a novel functional feature of the lectin chaperones and ERp57 binding for the folding and assembling of the multisubunit secretory protein fibrinogen in HepG2 cells. Notably, two-dimensional SDS-PAGE, in combination with non-reducing and reducing conditions, is a useful method for clarifying the assembly pathway of the multisubunit proteins especially in their early biosynthesis period combination with pulse-chase labeling as it was revealed that BiP and PDI preferentially bind to the hexamer while CNX associates with monomeric form of fibrinogen α and γ subunit [14]. Further study will be required for the construction of the entire ER chaperone assistance picture to support fibrinogen maturation including BiP, which binds to only β chains, not α and γ chains, in transfected COS cells [35].

## Materials and Methods

### Antibodies and Chemical Materials

Rabbit anti-fibrinogen antibodies were purchased from DAKO (for western blotting and indirect immunostaining) and ICN (for immunoprecipitation). Mouse monoclonal anti-PDI (MBL, Japan), mouse monoclonal anti-glyceraldehydes-3-phosphate dehydrogenase (GAPDH, from Ambion), mouse anti-HSP47 (stressgen), mouse anti-GM130 (Becton, Dickinson and Company) were purchased from the indicated companies, respectively. Secondary antibodies from Goat for indirect immunostaining, conjugated with Alexa488 or Alexa594, were obtained from Molecular Probes. Rabbit anti-ERp57 and anti-CNX were previously characterized and described [36], [37]. PNGase F and Endoglycosidase H were purchased from New England BioLabs Inc. Castanospermine (CAS, Wako, Japan), Pansorbin cells (Calbiochem), dithiothreitol (DTT, Sigma-Aldrich) and UDP-glucose (Nakalai, Japan) were obtained from the sources shown.

### Western Blotting

HepG2 cells were cultured in Dulbecco’s modified eagle medium containing 10% fetal calf serum at 37°C under 5% CO_2_-95% air conditions. The cells were lysed in SDS-PAGE sample buffer (10 mM Tris-HCl pH 8.8, 13% sucrose and 1% SDS) containing 1 mM iodoacetamide (IAA). After sonication and heat denaturing, the proteins were separated by SDS-PAGE and then transferred onto nitrocellulose membranes (Protoran, Schleicher & Schuell). After blocking by phosphate-buffered saline (PBS) containing 0.05% Tween-20 and 1% polyvinylpyrroridone (Sigma-Aldrich), the membranes were incubated with primary antibodies and then with horse-radish-peroxidase conjugated anti-rabbit or anti-mouse IgG (Pierce). Protein bands were visualized using an ECL system (GE Healthcare). For DTT treatment experiments, cells were exposed to various concentrations of DTT in culture medium for 10 min at 37°C. The free thiol group was alkylated by treatment of cells after the reaction with PBS containing 25 mM IAA on ice for 10 min before lysis.

### Metabolic Radiolabeling

HepG2 cells were cultured in a Met- and Cys-free medium (Invitrogen) for 3 hr and then pulse-labeled with [^35^S]Met and [^35^S]Cys labeling mix (GE Healthcare) at 37°C. In the chase experiments, the radiolabeled cells were further incubated with Dulbecco’s modified eagle medium containing 2.5 mM L-Cys, 5 mM L-Met and 0.2% bovine serum albumin. In the CAS treatment, the drug was added through starvation to the end of chase. For secretion assay (Fig. 2), the chase medium was replaced every 20 min. Radiolabeled cells were lysed in 2% Triton X-100 containing lysis buffer (10 mM Tris-HCl pH 7.4, 150 mM NaCl and 1 mM phenylmethylsulfonyl fluoride). Immunoprecipitation experiments were performed as previously described [7], [36]. Immunopurified proteins were washed and denatured in SDS-PAGE sample buffer by heating at 75°C for 10 min in the presence or absence of 50 mM DTT. Samples were resolved by SDS-PAGE with ^14^C molecular weight marker (GE Healthcare). Radioactive protein bands were visualized using BAS-2000 (Fujifilm, Japan).

### Two-dimensional SDS-PAGE

Proteins were first separated by non-reducing gradient SDS-PAGE (4–15%), and the corresponding lane was cut out. Proteins in the gel were reduced by heating in SDS-PAGE sample buffer containing 2% β-mercaptoethanol at 75°C for 20 min. Gel strips were installed on the top of 9% acrylamide gels and proteins were separated by SDS-PAGE. Radioactive proteins were visualized by a BAS-2000 image analyzer (Fuji film).

### Construction of Cyan Fluorescent Protein (CFP)-ERp57 Stably Overexpressing HepG2 Cells

cDNA of ERp57 was obtained from reverse-transcribed mRNA isolated from HepG2 cells using the primer 5′-GGTTTTCCATCTCTGATGGA-3′ and amplified by PCR using the primers 5′-GCCCGCCTCGTGTACACCTCCGACGTGC-3′ and 5′-GTGTTTGGCTTGTACATTAGAGATCCTCCTGTG-3′. The obtained cDNA was digested with BsrGI (New England BioLabs Inc) and subcloned into a pECFP-ER vector (Clontech). DNA fragments containing the CFP-ERp57 region were inserted into the pCX4-bsr vector, and the packaging cell (Phoenix Amp) was transfected with the vector. HepG2 cells were infected with retrovirus recovered from the media of packaging cells [38], [39]. A HepG2 cell line stably expressing CFP-ERp57 was selected by culturing in the presence of 10 µg/ml blasticidin.

### Indirect Immunostaining and Confocal Microscopy

HepG2 cells were split onto coverslips in 35 mm dishes before the experiment day. After fixation with 4% paraformaldehyde in PBS at room temperature for 10 min, membrane permeabilization was conducted using 0.1% TX-100 in the immunostaining buffer (PBS containing 5% glycerol and 1% Goat serum) at 2°C for 1 min. Cells were incubated with primary antibody: rabbit anti-fibrinogen, mouse anti-GM130 or mouse anti-HSP47 at 1∶100 dilution in the immunostaining buffer at room temperature for 20 min and then stained with 1∶400 Goat secondary antibodies: Alexa488 (fibrinogen) or Alexa594 (GM130 or HSP47). After rinsing with distilled water, coverslips were mounted face down on slide glasses with ProLongGold (Invitrogen). Images were taken with confocal laser microscopy (LSM 780, Carl Zeiss, Inc.) and processed with Photoshop software (Adobe).

### Microsomal Folding Assay

Fibrinogen folding assay using microsomes recovered from pulse-labeled HepG2 cells was performed essentially, as described previously [7].

### RNA Interference

RNAi target sequences and purified RNA duplexes, non-specific control (5′-GUA CCG CAC GUC AUU CGU AUC-3′) and ERp57 (5′-GGA UAA CUA CCG AUU UGC ACA-3′) were purchased from RNAi co. ltd., JAPAN. HepG2 cells were transfected with 5 nM double-strand RNA oligomers for 96 hr using LipofectAMINE 2000 (Invitrogen). The knockdown effect of ERp57 was verified by western blotting and quantified by ImageJ software (NIH) using GAPDH as a control.

## Supporting Information

Figure S1 **Western blotting analysis of fibrinogen molecules in HepG2 cells.** (A) HepG2 cells were lysed in SDS-PAGE sample buffer and heat denatured in the presence (Red) or absence (Non-Red) of 50 mM DTT. After SDS-PAGE in 9% gels, proteins were transferred to the membrane and visualized using anti-fibrinogen antibody. (B) Non-reducing gels were subjected to second reducing SDS-PAGE, as described in Fig. 1B, and immunoblotting using anti-fibrinogen antibody was performed. The molecular weight of marker proteins is indicated on the left in kDa, and fibrinogen molecules are shown on the right.
(TIF)

Figure S2 **ERp57 binds to fibrinogen trimer.** Starved HepG2 cells were pulse-labeled for 20 min and chased for 0 or 1 hr before immunoprecipitation, as described in the Materials and Methods, with anti-fibrinogen or anti-ERp57 antibodies (1st IP). The immunoisolated samples were denatured in 10 mM Tris-HCl (pH = 7.5) containing 150 mM NaCl and 1% SDS at 75°C for 10 min. After centrifugation, SDS concentration of the supernatant was diluted and re-immunoprecipitated with anti-fibrinogen antibody (2nd IP). Protein samples were resolved in SDS-PAGE in 4–15% gels under non-reducing conditions.
(TIF)
