# Supplementary figures and images for: Stepwise Assembly of Fibrinogen Is Assisted by the Endoplasmic Reticulum Lectin-Chaperone System in HepG2 Cells
Source: PLoS One. 2013 Sep 10;8(9):e74580. doi: 10.1371/journal.pone.0074580 (PMC3769264; doi:10.1371/journal.pone.0074580)

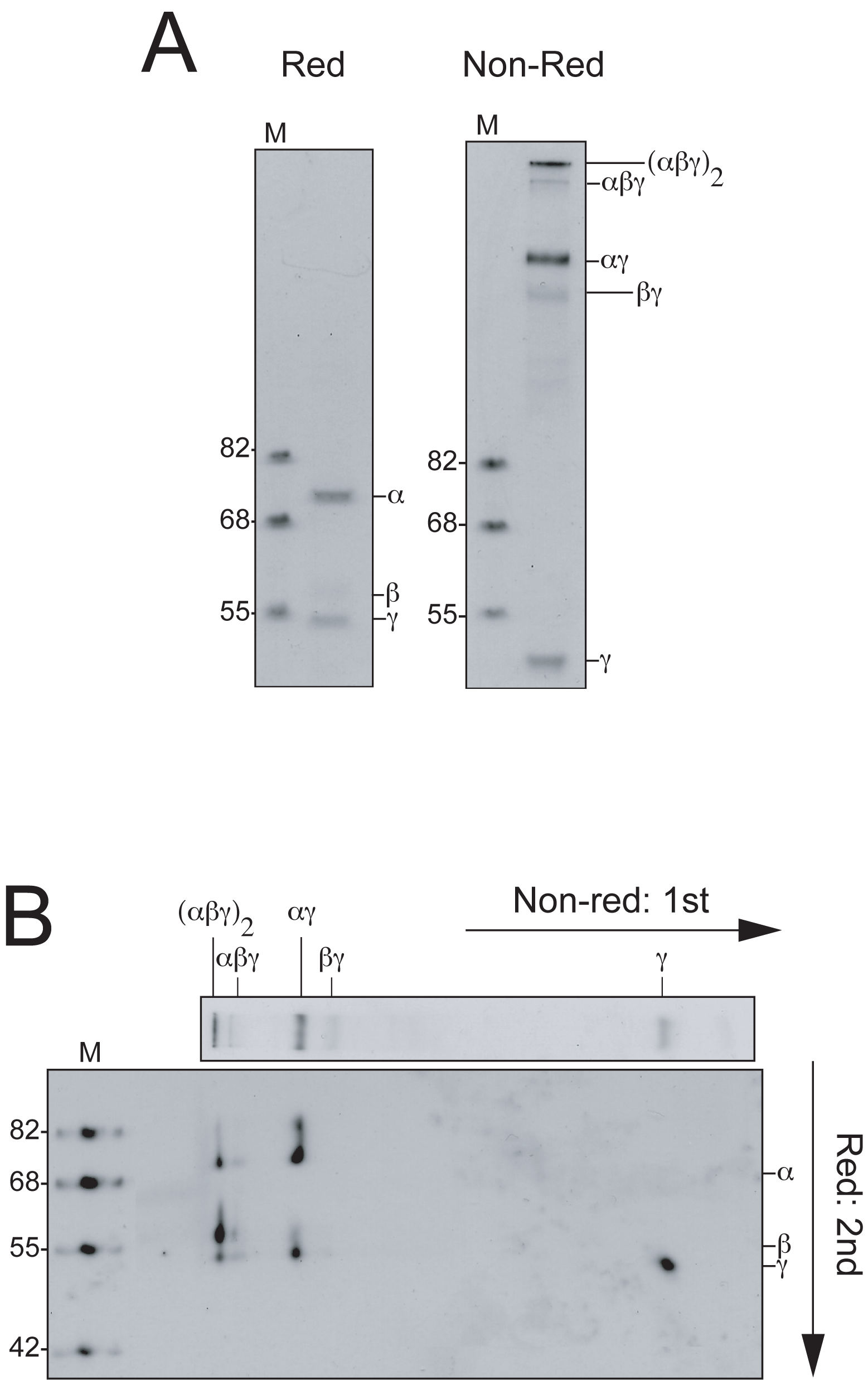

Supplement: Figure S1 — Western blotting analysis of fibrinogen molecules in HepG2 cells. (A) HepG2 cells were lysed in SDS-PAGE sample buffer and heat denatured in the presence (Red) or absence (Non-Red) of 50 mM DTT. After SDS-PAGE in 9% gels, proteins were transferred to the membrane and visualized using anti-fibrinogen antibody. (B) Non-reducing gels were subjected to second reducing SDS-PAGE, as described in Fig. 1B, and immunoblotting using anti-fibrinogen antibody was performed. The molecular weight of marker proteins is indicated on the left in kDa, and fibrinogen molecules are shown on the right. (TIF) [file pone.0074580.s001.tif]

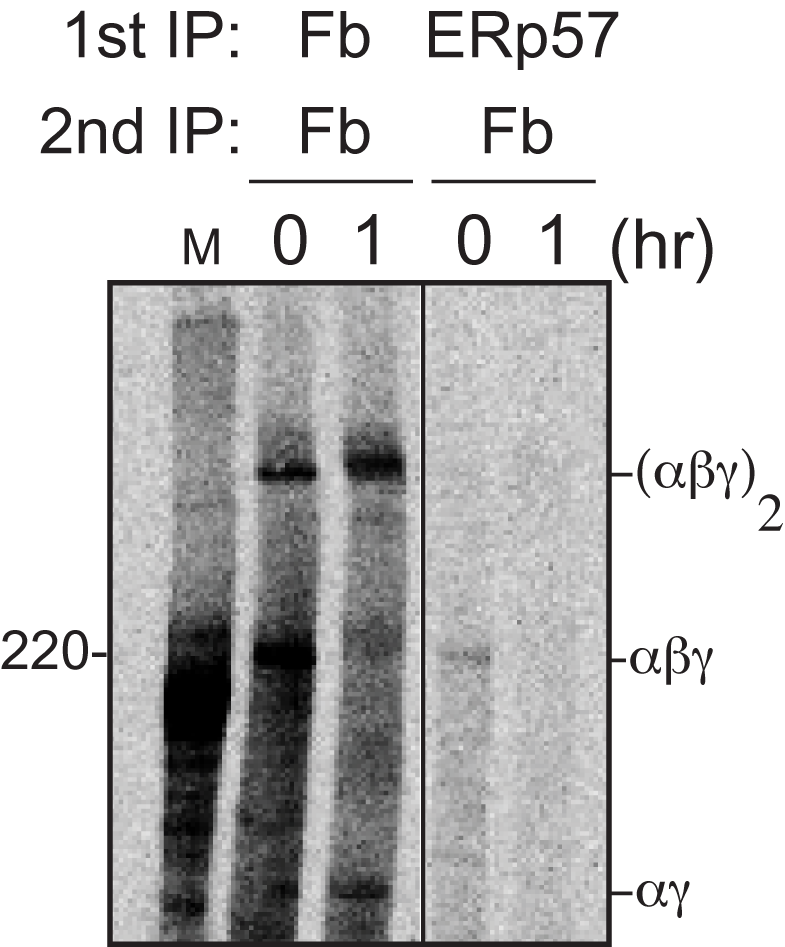

Supplement: Figure S2 — ERp57 binds to fibrinogen trimer. Starved HepG2 cells were pulse-labeled for 20 min and chased for 0 or 1 hr before immunoprecipitation, as described in the Materials and Methods, with anti-fibrinogen or anti-ERp57 antibodies (1st IP). The immunoisolated samples were denatured in 10 mM Tris-HCl (pH = 7.5) containing 150 mM NaCl and 1% SDS at 75°C for 10 min. After centrifugation, SDS concentration of the supernatant was diluted and re-immunoprecipitated with anti-fibrinogen antibody (2nd IP). Protein samples were resolved in SDS-PAGE in 4–15% gels under non-reducing conditions. (TIF) [file pone.0074580.s002.tif]
